# Using heterogeneity of the patient-derived xenograft model to identify the chemoresistant population in ovarian cancer

**DOI:** 10.18632/oncotarget.2373

**Published:** 2014-08-19

**Authors:** Zachary C. Dobbin, Ashwini A. Katre, Adam D. Steg, Britt K. Erickson, Monjri M. Shah, Ronald D. Alvarez, Michael G. Conner, David Schneider, Dongquan Chen, Charles N. Landen

**Affiliations:** ^1^ Division of Gynecologic Oncology, Department of Obstetrics and Gynecology, University of Alabama at Birmingham; ^2^ NIH Medical Scientist Training Program, University of Alabama at Birmingham; ^3^ Department of Pathology, University of Alabama at Birmingham; ^4^ Department of Biochemistry and Molecular Genetics, University of Alabama at Birmingham; ^5^ Division of Preventative Medicine, Department of Medicine, University of Alabama at Birmingham; ^6^ Division of Gynecologic Oncology, Department of Obstetrics and Gynecology, The University of Virginia, Charlottesville, VA

**Keywords:** Ovarian Cancer, Patient-derived xenograft, cancer stem cells, chemoresistance, animal models of cancer

## Abstract

A cornerstone of preclinical cancer research has been the use of clonal cell lines. However, this resource has underperformed in its ability to effectively identify novel therapeutics and evaluate the heterogeneity in a patient's tumor. The patient-derived xenograft (PDX) model retains the heterogeneity of patient tumors, allowing a means to not only examine efficacy of a therapy, but also basic tenets of cancer biology in response to treatment. Herein we describe the development and characterization of an ovarian-PDX model in order to study the development of chemoresistance. We demonstrate that PDX tumors are not simply composed of tumor-initiating cells, but recapitulate the original tumor's heterogeneity, oncogene expression profiles, and clinical response to chemotherapy. Combined carboplatin/paclitaxel treatment of PDX tumors enriches the cancer stem cell populations, but persistent tumors are not entirely composed of these populations. RNA-Seq analysis of six pair of treated PDX tumors compared to untreated tumors demonstrates a consistently contrasting genetic profile after therapy, suggesting similar, but few, pathways are mediating chemoresistance. Pathways and genes identified by this methodology represent novel approaches to targeting the chemoresistant population in ovarian cancer

## INTRODUCTION

Although most ovarian cancer patients present with advanced-stage disease, response to front-line platinum-based chemotherapy is high, on the order of 75%. The combination of surgery and adjuvant chemotherapy will allow remission in most patients, and about 40% of advanced stage patients will live at least 5 years [1]. However, absolute cures are uncommon, with 80% of patients eventually having a recurrence [2]. The clinical profile of high rates of positive responses yet high recurrence rates suggests the presence of a subpopulation of cells within the heterogeneous tumor that survives initial chemotherapy, to lie dormant and eventually regrow with chemoresistant disease. Only by targeting this subpopulation can we achieve durable cures [3, 4].

Pre-clinical models used in drug discovery have predominately used clonal ovarian cancer cell lines, which cannot account for tumor heterogeneity, and evolve though selective growth and time to become very different from tumors growing in patients. Recently some of the most commonly used ovarian cell lines used were reported to have profiles more like endometrioid than papillary serous carcinoma, as defined by TCGA expression profiling[5]. Studying tumors preclinically that more closely resemble human tumors may increase the likelihood that medications effective in preclinical studies are effective in clinical trials. The patient-derived xenograft (PDX) model, whereby tumors are collected from patients and immediately implanted into mice, has recently been characterized and may allow such an advantage[6-8].

We set out to further characterize the PDX model and determine whether the heterogeneity seen in ovarian cancer is recapitulated, in order to explore the cell populations responsible for chemoresistance. One potential subpopulation with chemotherapy resistance is the cancer stem cell (CSC) population. CSC's have been shown to have increased tumorigenicity in mice, chemotherapy resistance, and are enriched in recurrent ovarian cancer [9-11]. In developing and characterizing the PDX model our goals were to 1) optimize methods to allow a high success rate of implantation, 2) examine retention of heterogeneity, 3) determine if PDX tumors respond to chemotherapy similarly to patient tumors, 4) assess whether treatment with chemotherapy results in survival of just CSC populations, and 5) identify pathways that are amplified in resistant tumors. We demonstrate that the PDX model can be established with a high success rate, have similar expression profiles and biologic activities as patient tumors, and can be used as a model to identify the chemoresistant population.

## RESULTS

### Implantation success rate and establishment of the ovarian PDX model

Here we report outcomes on the first 34 patient samples implanted into SCID mice. Demographics for patients from whom tumors were collected are presented in Table 1. All patients had stage IIIC or IV high-grade epithelial ovarian cancers, and tumors were collected prior to any chemotherapy.

Tumor collected and implanted into mice was either from an omental metastasis or peritoneal implant, since they are plentiful, composed of grossly-identifiable tumor, and most relevant to recurrent disease.

**Table 1:** Patient demographics of implanted and growing patient-derived xenograft (PDX) lines

Characteristic			Percent or Average (range)
Age at diagnosis			61.7 (47-87)
Stage			
		Stage IIIC	83%
		Stage IV	17%
Race			
		Caucasian	76%
		African American	24%
Procedure			
	Tumor Reductive Surgery	Optimal TRS	25%
		Suboptimal TRS	45%
	Laparoscopic Biopsy prior to neoadjuvant chemotherapy		3%
Histology			
		Papillary Serous Adenocarcinoma	79%
		Endometroid	3%
		Mixed Epithelial	9%
		Mucinous	3%
		Extra-ovarian in origin	6%
Chemotherapy Treatment			
		Carboplatin	4%
		Carboplatin/Avastin	4%
		Carboplatin/Paclitaxel	56%
		Carboplatin/Paclitaxel/Avastin	7%
		Carboplatin/Taxotere	19%
		Cisplatin/Docetaxel	4%
		Cisplatin/Paclitaxel	4%
		Cisplatin/Taxotere	4%

**Figure 1:**
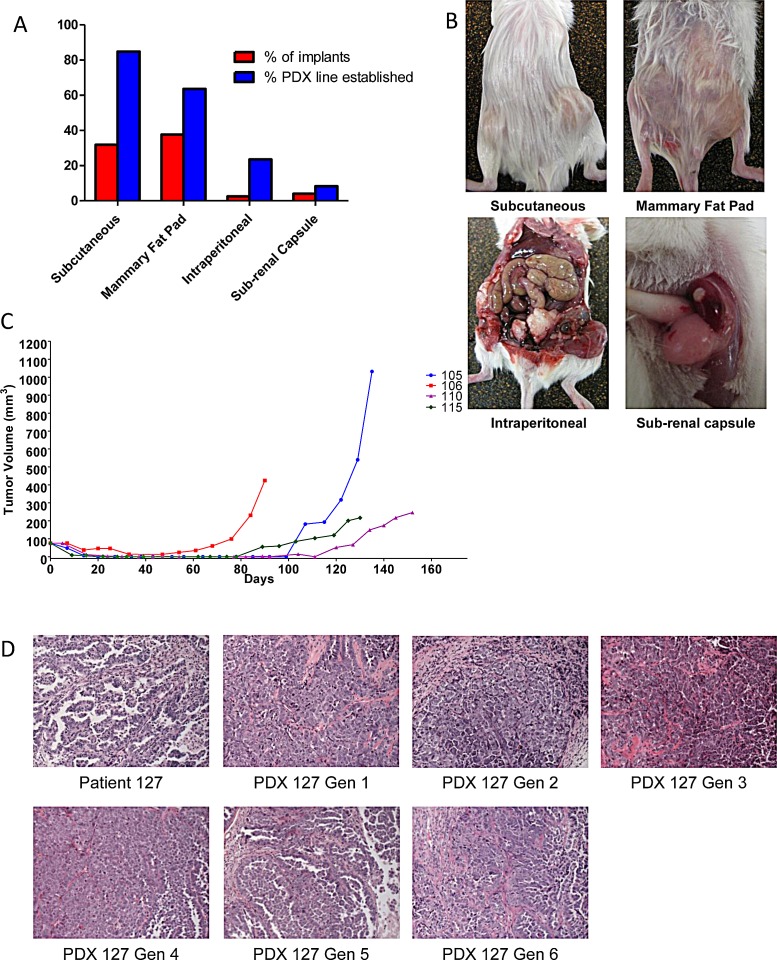
Take rates of different sites for implantation and maintenance PDX histology (A) Tumors were implanted subcutaneously (SQ), in the mammary fat pad (MFP), intraperitoneal (IP), or sub-renal capsule. The success of implantation was similar comparing SQ to MFP, however more PDX lines were established from SQ implant due to number of implants. IP and SRC implants are not effective for establishing a PDX line. (B)Representative pictures of implanted tumors at either SQ, MFP, IP, or SRC. (C) After implantation, tumor volume decreased to an undetectable size then re-grew after a dormancy period. This implicate the small population of tumorigenic cells survive and re-capitulate the tumor after implantation. Representative growth chart showed of 4 different PDX lines after implantation. (D) Histology of the original tumor is maintained throughout subsequent generations. Patient 127 had a histology of papillary serous adenocarcinoma that has been maintained for 6 generations in the corresponding PDX.

Different sites of implantation in the mouse were tested to identify the best location for growth. Subcutaneous (SQ) and mammary fat pat (MFP) sites were tested as their location allows for tumor growth to be monitored with caliper-measurements. Intraperitoneal (IP) injection was examined, to provide an orthotopic location for model establishment. The subrenal capsule (SRC) was evaluated given previous reports of high take rates in this site [12]. Implantation for all 4 sites was conducted as described in the methods. Therefore both site and method of processing were controlled for each patient. The rates for PDX tumor development in each site, including individual implants are presented in Figure 1A. In the first 34 patients, a PDX line was established in 85.3% of SQ implants. This is compared to 63.64% in the MFP, 22.2% IP, and 8.3% in the SRC. SQ xenografts almost always visually disappeared in the weeks after implantation before regrowing and being detectable at a mean of 78.4 days after implantation (range 17-174 days, Figure 1C) compared to 77.3 days for the MFP (range 29 to 129 days, NS). The success of a PDX being established is highest in the SQ site in part due to the increased number of implants per patient. Based on this data, and subsequent studies showing similar expression profiles in tumors from the SQ site and original patient tumors (described below), continued development of the PDX model was done in the SQ site. PDX tumors were examined for histologic characteristics by a gynecologic pathologist. In all cases and in up to six generations of reimplantation, the original histology was maintained (Figure 1D). Interestingly, in the few cases where a mixed epithelial-type ovarian cancer was implanted, both histologies were present in each of the subsequent PDX generations.

### Heterogeneity of PDX tumors

**Figure 2:**
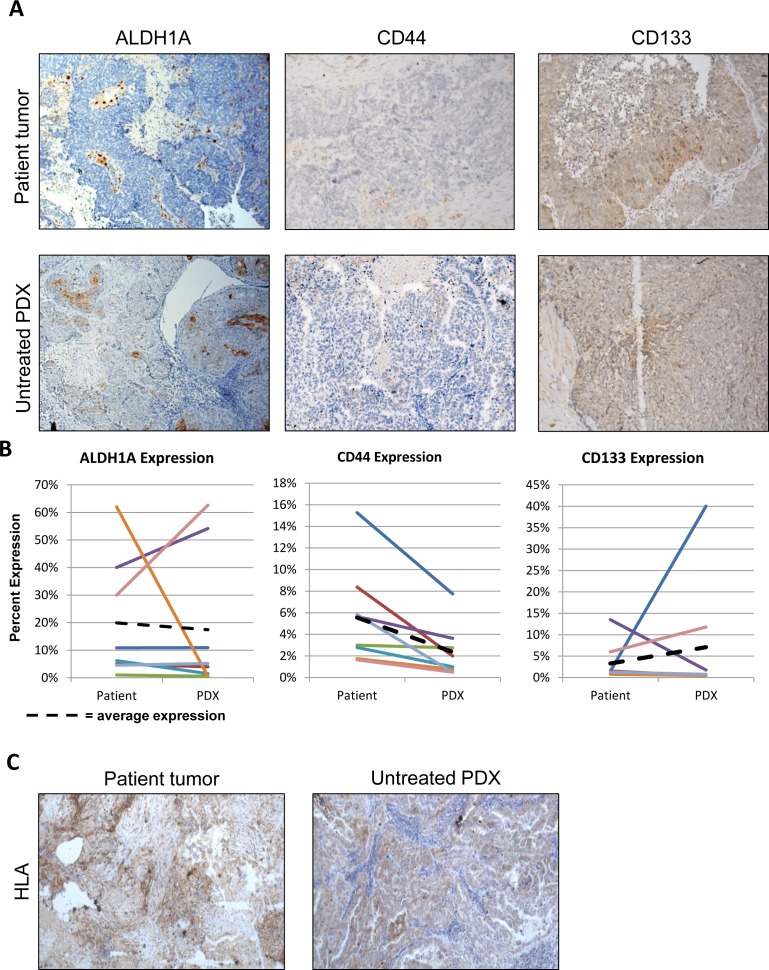
Establishment of the PDX line does not enrich for the tumorigenic cell population and human stroma is replaced in the implanted PDX (A) Representative staining for ALDH1A1, CD133, and CD44 on the patient sample and untreated PDX. (B) Quantification of change in expression of ALDH1A1, CD133, and CD44 between the patient sample and the untreated PDX. Only CD44 had a significant decrease in expression (p-value <0.05). ALDH1A1 and CD133 had no significant change in expression. (C) Human HLA expression in patient and untreated PDX tumors, demonstrating replacement of human stroma with murine cells.

One potential advantage of the PDX model is that it may maintain patient heterogeneity, as opposed to the clonality that ultimately characterizes cell lines. However, a growing body of evidence suggests that certain cell subpopulations have enhanced ability to initiate tumors, often termed tumor-initiating cells (TIC's) or sometimes CSC's if additional attributes are demonstrated [10]. We examined whether resulting PDX tumors maintained tumor heterogeneity from a tumor-initiating cell standpoint. PDX tumors and original patient tumors were subjected to IHC for the TIC markers ALDH1A1 [11, 13, 14], CD133 [15-17], and CD44 [18, 19]. For ALDH1A1, CD44, and CD133, the patient samples averaged expression of 19.95%, 5.56%, and 3.27% respectively. The PDX tumors had similar expression of ALDH1A1 and CD133 at 17.4%, and 7.1% respectively (p=0.80 and 0.49, Figure 2A, 2B). There was a significant change in expression of CD44, but it was actually a decrease, from 5.54% to 2.36% (p=0.014). If TICs in ovarian cancer are indeed the cells mediating xenograft formation, these data suggest that they subsequently differentiate into marker-positive and -negative cells and recapitulate tumor heterogeneity, in keeping with the CSC hypothesis[10, 20].

Related to heterogeneity, the human/murine component of PDX tumor would have implications to the biologic relevance of this model. IHC for human HLA antigen was conducted to identify the species-specific composition of the PDX tumor. Interestingly, all stromal cells in the PDX tumors were of murine origin (Figure 2C). This was consistent across 100% of the tumor specimen, and in all of the first 15 PDX tumors established.

### Biological and clinical characterization of PDX tumors

**Figure 3:**
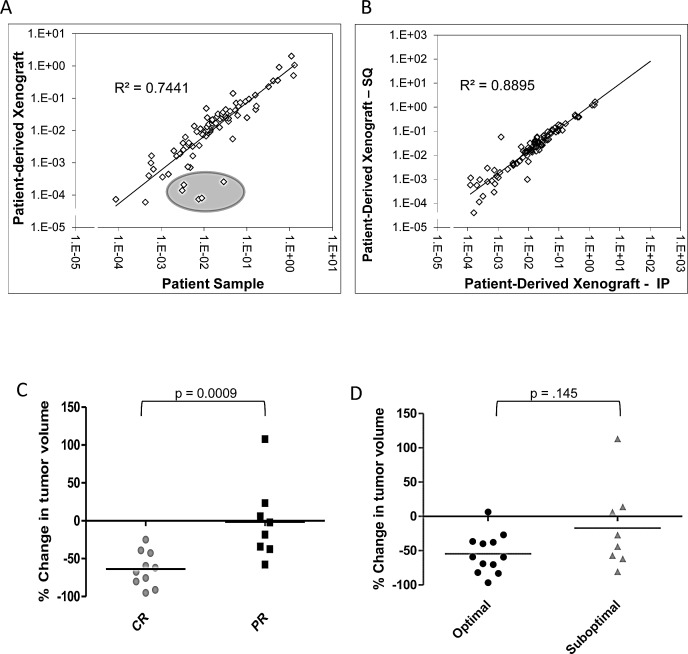
Cancer drug targets are maintained in the PDX line and the PDX response to treatment correlates to the patient's response to primary chemotherapy (A) The SABiociences RT^2^ qPCR array for cancer drug targets was run on the patient's tumor and their matched untreated PDX tumor. Differences in relative gene expression for each target was calculated and the 2^ΔCt^ value was determined. Most of the 84 cancer drug target genes had similar expression in the PDX and the original patient sample. 5 gene were down-regulated in the PDX sample, though all 5 are related to VEGF and PDGF signaling (circled in grey). (B) The SABiosciences RT^2^ qPCR array for cancer drug targets was run on matched subcutaneous PDX tumors and intraperitoneal PDX tumors. Differences in relative gene expression for each target was calculated and the 2^ΔCt^ value was determined. All 84 cancer drug target genes showed a strong correlation between the IP and SQ PDX tumors(C) PDX lines were treated with combination carboplatin and paclitaxel IP weekly. The percent change in tumor volume at 30 days was compared to the patient's response to primary therapy. PDX lines with the greatest decrease in volume significantly correlated to patients with a complete response to therapy (p=0.0009) (D) Classifying reduction in tumor volume by outcome of tumor reductive surgery (optimal debulking vs suboptimal) shows a trend towards PDX with the greatest reduction in volume correlating to optimal debulking for the patient (p-value = NS).

To begin to evaluate the biologic characteristics of PDX tumors compared to original patient tumors, we examined oncogenic expression, proliferation, and response to chemotherapy. Weroha *et al* have previously demonstrated similar amplification and deletion patterns between PDX and patient tumors using aCGH [6]. To characterize whether expression of key oncogenes are similarly expressed in PDX tumors, an RT^2^ PCR array on four pair of patient samples and matched PDX tumors was used. This array quantifies mRNA levels of 84 genes that are recognized targetable oncogenes[21]. There was a strong correlation of expression in 79 of the cancer drug targets, with an overall R^2^-value of .744 (Figure 3A). This correlation was also present in individual samples (Supplemental Figure 1). The five genes that exhibited the poorest correlation had expression in the patient with near-zero mRNA expression in the PDX. These genes were platelet-derived growth factor receptor, alpha and beta polypeptide (PDGFRA, PDGFRB) and vascular endothelial growth factor receptor one, two, and three (VEGFR1, VEGFR2, VEGF3). These genes were expected to be decreased in the PDX tumor, since they are produced by the host, and the primers are human-specific. Therefore, there is strong consistency in expression of targetable oncogenes intrinsic to malignant cells, despite the fact that these tumors are growing in the subcutaneous compartment. In addition, we profiled the genetic difference of oncogene expression using the RT^2^ PCR array comparing PDX tumors from the IP location versus the SQ implant. There was a strong correlation of expression among the 84 genes in the oncogene drug target array, with an overall R^2^-value of .8895 (Figure 3B). This indicates that the SQ tumor has similar expression to a tumor growing in the orthotopic location.

While expression at the single-gene level is important, biologic similarity regarding response to treatment is equally important. Mice with measurable tumors from 19 PDX models were treated with IP carboplatin (90 mg/kg/week) and paclitaxel (20 mg/kg/week) in combination for 4 weeks. After 4 weeks, percent-reduction in tumor volume was calculated and compared to the patient's response to therapy, categorized as complete (CR, no evidence of disease at completion of 6 cycles of primary chemotherapy) or partial (PR, residual disease present at completion of 6 cycles of primary therapy). Patients that had a CR to therapy had an average reduction in volume of 63.73% (range 95.04% to 24.87%) compared to an average reduction of just 1.53% (range 57.77% reduction to 107.9% increase) in patients that had a PR (p = 0.0009, Figure 3C). There was also a differential, but not significant, response between patients who had an optimal or suboptimal tumor reductive surgery (Figure 3D). While not definitive, this suggests that patients presenting with disease unable to be optimally debulked are more aggressive and resistant to chemotherapy.

### Biologic mediators of chemotherapy resistance in the PDX

**Figure 4:**
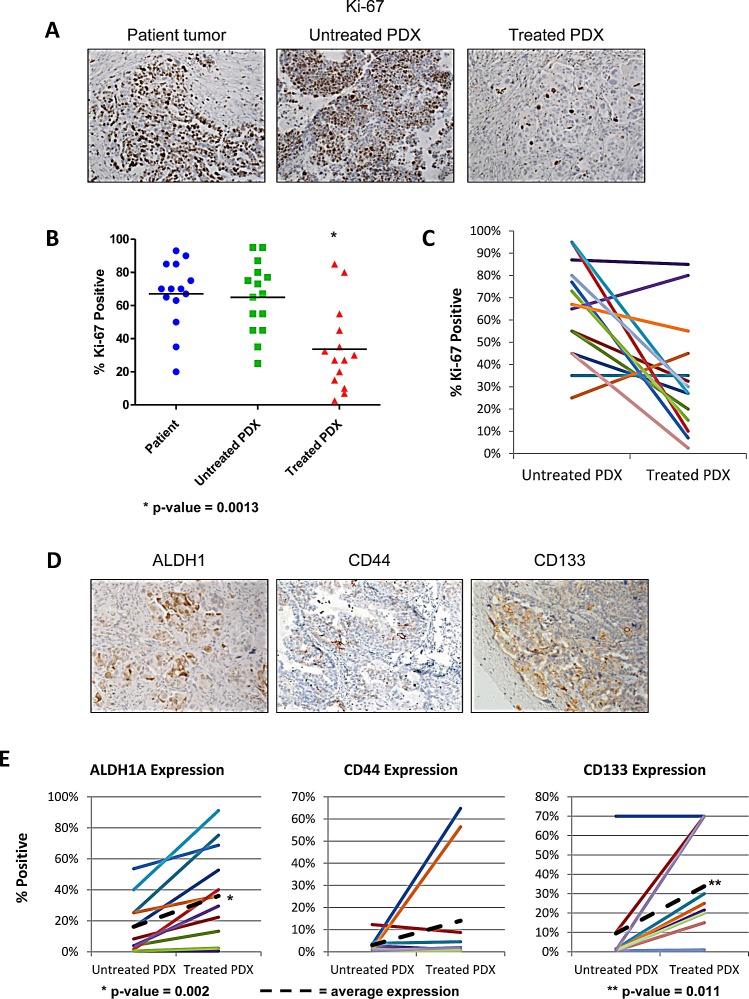
Chemotherapy treatment reduces proliferation and enriches the PDX for cancer stem cells Tumor cell proliferation was quantified using the Ki67 marker on original patient samples, untreated PDX samples, and chemotherapy treated PDX samples. Change in cancer stem cell marker expression was analyzed after chemotherapy treatment. (A) Representative IHC of Ki67 staining in the patient sample, untreated PDX, and treated PDX. (B) On average, proliferation decreases with chemotherapy treatment in all PDX lines tested. There is no significant change in proliferation between the patient and the untreated PDX. (C) Proliferation rates for each treated and matched untreated pair show that the majority of tumors have a reduced proliferation rate after chemotherapy treatment (D) Representative IHC of CSC markers ALDH1A1, CD133, and CD44 of PDX treated with carboplatin and paclitaxel for 4 weeks. (E) In the treated PDX, expression of ALDH1A1 and CD133 are significantly increased (p-value = 0.0023 and p-value = 0.011 respectively).

With evidence showing that the PDX model accurately replicates the biology and clinical properties of the original patient tumor, we sought to explore differences between matched untreated and treated tumors. Mice were treated as described above, with tumors harvested 6 days after the 4^th^ weekly dose, to minimize acute tumoral effects that might occur after chemotherapy administration. Ki-67 was examined to measure proliferation, and was not significantly different in untreated PDX tumors compared to the original patient tumor (Figure 4A,B). However, treated tumors had significant decrease in Ki-67 positivity (33.6% compared to 64.9% in untreated tumors p=0.0013). Examining the trend of each tumor individually (Figure 4C), two pair actually showed an increase in Ki-67, one of which had a 107% increase in tumor size on therapy, but the other with a 70.9% reduction. Despite these aberrations, the overall decrease in proliferating fraction suggests that dormancy is either being induced by chemotherapy, or some cells are already in a dormant state at presentation, and have intrinsic resistance to chemotherapy.

For analysis of which subpopulations have enhanced survival with chemotherapy, we assessed the density of the CSC populations expressing ALDH1A1, CD44, and CD133. If these populations were important to survival in the presence of chemotherapy, they should be more densely present after treatment, as noted in human specimens [11]. Treatment resulted in the significant enrichment of ALDH1A1-positive cells (increased from 16.2 to 36.1%, p=0.002) and CD133-positive cells (increased from 9.5% to 33.8%, p=0.011) (Figure 4D). Mean CD44 expression increased, but this was driven by two samples, and was not significant. These data suggests treated tumors are enriched in CSC populations.

### Differential expression of genes due to chemotherapy treatment

**Figure 5:**
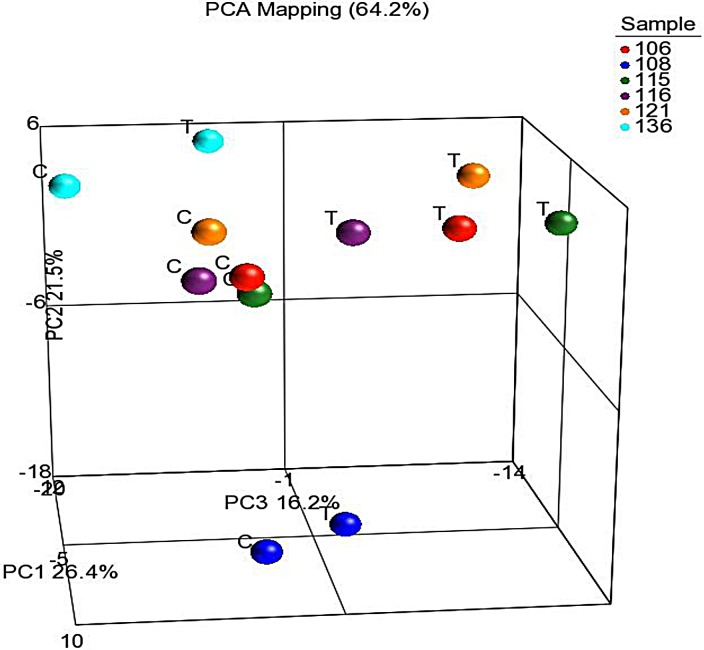
RNAseq comparing the treated PDX lines to the untreated PDX lines Principal component analysis of genes expression in the treated and untreated PDX tumors. While matched treated and untreated PDX tumors clustered together, most treated PDX tumors had change of expression in the same direction indicating a small subset of genes responding to chemotherapy.

Although cells with CSC properties were increased in treated specimens, they did not make up the entirety of the tumor. To globally examine which other genes and pathways are significantly altered during chemotherapy treatment, RNA-Seq was conducted on 6 pairs of treated and untreated PDX tumors. Across all six pairs, 299 genes were found to be significantly differentially expressed in the treated PDX samples compared to untreated (Supplementary Table 1), 137 of which have known roles in cancer. The top up-regulated genes and down-regulated genes are in Table 2. When principal component analysis was performed, an interesting trend emerged. Four of the samples clustered together, and the remaining two were separated in the 3D space. All the treated samples showed a shift in the same direction away from their untreated PDX pair (Figure 5). This indicates that while the majority of genes are similar before and after treatment, all six tumors were affected similarly by therapy. IPA pathway analysis identified 5 major pathways that were significantly altered with treatment and key changes in molecular and cellular function (Table 2). Changes in these biological functions and pathways are consistent with the visualized phenotype of tumors responding to chemotherapy and reorganizing cellular function to adapt for survival.

**Table 2:** RNAseq analysis on PDX comparing 6 pairs of treated versus untreated samples

Top Canonical Pathways	P-Value
Protein Kinase A Signaling	3.58E-05
GNRH Signaling	2.74E-04
Sphingosine-1-phosphate signaling	5.4E-04
α-Adrenergic signaling	9.39E-04
Cholecystokinin/Gastrin-mediated signaling	1.91E-03
**Molecular and Cellular Functions**	
Lipid Metabolism	1.33E-04 to 3.80E-02
Molecular Transport	1.33E-04 to 3.80E-02
Small Molecule Biochemistry	1.33E-04 to 3.80E-02
Cell Morphology	1.64E-04 to 3.80E-02
Cellular Assembly and Organization	1.64E-04 to 3.75E-02
**Top Up-Regulated Molecules**	Fold-Change Expression
ZNF750	2.441
ACP5	2.294
HIST2H2BE	2.141
CPEB3	2.117
DNM3	2.028
MPC1	1.980
ABCG1	1.938
MGLL	1.924
TLR5	1.884
**Top Down-Regulated Molecules**	Fold-Change Expression
APOC1	-2.488
GPHA2	-2.262
POLR3G	-1.862
TES	-1.759
PLCE1	-1.738
PUS7	-1.618
ARNT2	-1.607
MECOM	-1.570
CKAP4	-1.564
KLF5	-1.554

## DICUSSION

We demonstrated the feasibility of an ovarian PDX model that closely models the heterogeneity of the original patient's tumor and maintains clinical relevance. Ovarian PDX tumors form at a high rate when placed in the subcutaneous location. Growing tumors recapitulate the heterogeneity of the original patient tumor, and are not composed of just TICs, though the stromal component is murine. The PDX tumors have similar oncogene expression as the patient tumor, and respond to chemotherapy in a similar manner as the patients from which they were harvested. These similarities make the PDX model an attractive platform for pre-clinical testing of therapies that will hopefully correlate with a clinical response better than noted in cell lines. Finally, using this model has allowed identification of pathways mediating survival after chemotherapy that are attractive targets for future study.

In most malignancies, preclinical studies have primarily utilized cell lines to assess novel therapies and biologic processes. Cell lines are still ideal for carefully controlled studies on mechanisms and pathways. However, in terms of translating results to the clinic, these models have underperformed [22]. The clonal nature of cell lines limits the ability to study both intratumoral and interpatient heterogeneity [8, 23]. In addition, new genomic studies indicate that commonly-used ovarian cancer cell lines do not accurately represent high-grade serous ovarian cancers when compared to profiling performed on the TCGA dataset[5 ].

Development of PDX models have been demonstrated in a few malignancies, including ovarian, colorectal, medulloblastoma, pancreatic, breast, and non-small cell lung cancers [6, 24-29], and have consistently been found to be similar to patient samples. One well-established program in pediatric malignancies has demonstrated prediction of response in the clinic is higher when the PDX model is used [30]. However, there are drawbacks to the model. The time for PDX tumors to grow is variable, but usually on the order of months, making experiments slow and expensive. Historically, rates for success of PDX establishment have been low, with the most successful models having 37% establishment rate [28, 31, 32], until Weroha's recent report of 74% overall success in ovarian cancer[6]. In this study, we had 85.29% success rate of establishing a PDX in the first 34 patients we implanted in the SQ. We believe the higher success rate is due to several factors. Given similar success of Weroha's report, this may be disease-specific. Strong working relationships with clinicians and pathologists allow for implantation within one hour of removal. We used two different processing methods that could be directly compared - one where solid tumors were implanted (SQ and SRC), and one where tumors were dissociated (MFP and IP). With both methods, the take rate was more dependent on the site implanted than the processing method. A crucial factor is the starting material. Other groups have reported that higher engraftment rates are associated with more aggressive tumors [6, 8, 29]. Instead of using the primary tumor from the ovary, we have implanted omental or peritoneal metastatic implants. The reasons for this are both biologic and practical. From a practical standpoint, omental implants are easily distinguished from normal tissue, reducing the risk of implanting normal tissue. A portion of “tumor” taken from the ovary, a complex tissue with normal solid components, may more likely be misinterpreted grossly as tumor, when in fact was benign. Because the omentum is well-vascularized, tumors are very “healthy”, giving additional confidence that the portion implanted is not necrotic. Finally, it has been demonstrated that other factors produced in the omental microenvironment are pro-tumorigenic, and are likely implanted with these tumors[33]. The biologic rationale for using metastatic implants is that these sites are more relevant to the portions of tumors that recur. Therefore it may be more clinically relevant to characterize the metastatic site.

The site of implantation is an important consideration as there are benefits and drawbacks from using an orthotopic or heterotopic site. Heterotopic locations allows for easier monitoring of the tumor while orthotopic preserves the appropriate microenvironment [24]. However, in developing this model, use of the intraperitoneal orthotopic location had practical limitations of lower engraftment rates and difficulty in assessment of growth. In several instances mice become moribund with ascites before there was appreciable tumor volume, even when following with micro-CT imaging. This limits the ability to measure response to a therapy, and provides less tissue for analysis and propagation into the next generation of PDX. However, the Weroha study demonstrated an ability for high take rate using the intraperitoneal injection with large volumes of tumor-cells [6]. Like our study, their mice also demonstrated development of ascites but by using ultrasound, were able to more accurately follow tumor progression then using a micro-CT. By using the heterotopic location, tumor growth can be easily monitored for establishment, growth, and response to therapy [8]. However, biologic relevance has to be demonstrated. With our findings that subcutaneous tumors have similar oncogene expression profiles to patient tumors and the orthotopic intraperitoneal PDX tumors, and respond to chemotherapy similarly, the subcutaneous model appears relevant. This information helps alleviate the primary concern of not using the orthotopic location and provides a mechanism for decreasing the technical complexity of establishing and using a PDX model. While in our hands, not enough intraperitoneal tumors developed to evaluate their correlation to the clinical response, based on our oncogene data comparing SQ and IP tumors and the Weroha study, it appears both models are equivalent. Not enough intraperitoneal tumors developed to demonstrate whether they would be equivalent, or superior, to the subcutaneous model. While previous groups have reported a high rate of success using the subrenal-capsule for tumor establishment [12], we did not see these successes. The ultimate proof of the importance of location in the PDX model will require testing numerous compounds, and relating the response in PDX tumors to responses in patients. PDX models in other malignancies have demonstrated a similar response rate between mice and the corresponding clinical trial [34-36]. Such studies in ovarian cancer are ongoing. But our analysis of the oncogene expression profiles, and their consistent similarity to patient tumors (Figure 3A), suggest that differences in targetable oncogenes between orthotopic PDX tumors and patient tumors are minimal.

We also demonstrate that the ovarian PDX model maintains the heterogeneity of the original patient tumor, at least from a TIC standpoint. Studies of CSC and TIC populations have shown that some cells are more capable of forming xenograft tumors than other[37]. Our analysis of density of ALDH1A1, CD44, and CD133 cells, the most consistent markers of TICs in ovarian cancer, demonstrates that PDX tumors are not only composed of these subpopulations (Figure 2B). It is possible that these subpopulations are the drivers of tumor formation, but as they grow they produce differentiated tumors with both CSC and non-CSC populations. This in fact would be predicted by the CSC model.

Potential limitations to the PDX model in ovarian cancer have been identified through our analysis. We saw that of 84 oncogenes examined, 5 were under-expressed in PDX tumors: receptors for platelet-derived growth factors and VEGF receptors. The fact that all members of these receptor families strengthens the validity of the association. Analysis of the species making up tumor stroma showed it to be composed purely of murine origin. The reduced content of human stromal genes is expected [38] as a result of the replacement of the human stroma with mouse stromal cells after implantation. Prior reports in pancreatic cancer have suggested that human stromal cells are maintained for several generations[39], although Weroha *et al* also found that IP ovarian PDX tumors had murine stroma. Whether murine stroma impacts the validity of the model will depend on the specific agent and pathway targeted.

The heterogeneity demonstrated in ovarian PDX tumors makes it uniquely positioned to investigate the key clinical problem of chemoresistance and recurrence. Ovarian cancer has a high rate of response to primary chemotherapy followed by an equally high rate of recurrence. One hypothesis is that this population is the same as the tumorigenic CSC population. While we have seen an increase in CSC density in the treated PDX tumors, and previously in treated patients[11], the persistent/recurrent tumors were by no means completely composed of these populations. Either the CSC populations had already begun to give rise to repopulating daughter cells negative for the CSC marker, or (more likely) other chemoresistant populations exist that cannot be identified by ALDH1A1, CD44, or CD133 alone. Going beyond CSCs, we have shown that surviving tumors have more cells in dormancy, decreasing from a baseline of 65% to 34%. RNA-seq analysis resulted in 299 genes being significantly different between the treated and untreated tumors with principal component analysis indicating that the changes in gene expression represent a small subset of the entire genetic makeup of the tumor (Figure 5, Supplementary Table 1). Most remarkable and encouraging is that the changes were similar in all pairs tested, providing hope that there may be common pathways to be targeted in most patients. One of the top up-regulated genes was ABCG1 (BCRP1), a member of the White family of ATP-Binding cassette (ABC) transporters. Expression of ABCG1 has been shown to identify a side population of cancer cells that demonstrate CSC properties and chemoresistance [40]. Interestingly, one of the top activated pathways identified by IPA Ingenuity pathway analysis was Sphingosine-1-phosphate signaling. This pathway has been shown to protect oocytes from apoptosis induced by chemotherapeutic agents *in vitro* and *in vivo* [41, 42]. Taken together, the enrichment of CSC markers in the treated population, decrease in cell proliferation, and increase in genes and signaling pathways predicted to play a role in chemoresistance, it appears that treatment of the ovarian PDX results in the survival of a cell population that is chemoresistant to primary therapy. The global analysis by RNAseq provides a snapshot of possible pathways that are responsible for the development of chemoresistance. These will be important targets for therapy in future studies. With the development of an ovarian PDX model that recapitulates the clinical response and the heterogeneity of ovarian cancer, investigators are positioned to more effectively evaluate novel therapeutics and use the model to improve our understanding of the mechanisms of chemotherapy resistance. Hopefully targeting these pathways will sensitize cells to chemotherapy and lead to more durable cures.

## CONCLUSION

Development of an ovarian PDX model to study *de novo* chemotherapy resistance provides a unique use of the xenograft model beyond testing pre-clinical compounds, allowing for possible novel understandings of tumoral responses to therapy that may lead to new strategies for targeting the residual survival population after primary therapy. 

## MATERIALS AND METHODS

### Collection and Implantation of tumor specimens

Under IRB and IACUC approval, patients with suspected ovarian cancer that were being treated by the Division of Gynecologic Oncology at UAB were consented for this study. At the time of primary tumor reductive surgery, a specimen from an omental metastasis or peritoneal implant that was not required for pathologic diagnosis was collected and transported to the laboratory for processing. Specimens were sectioned and a portion submitted for formalin-fixed-paraffin embedding; placed in RNAlater (Qiagen, Frederick, MD); snap frozen in liquid nitrogen, and slow freezing in Optimal Cutting Temperature (OCT) Medium, and stored at -80°C. Remaining tumor was isolated for implantation into SCID mice (NCI-Frederick, Frederick MD) into four sites: subcutaneous (SQ), subrenal capsule (SRC), intraperitoneally (IP), and mammary fat pad (MFP). To discover the optimal site for tumor growth, of the first 22 patients, 22 were implanted SQ and MFP, 18 IP, and 12 SRC. When enough tumor was available, all four sites were implanted to allow direct comparison of growth rates. After it was evident that the subcutaneous implantation site was optimal, an additional 11 patients had tumors implanted only SQ.

For SQ implants, 5mm^2^ tumor pieces (n=20 per patient) adjacent to the slice used for confirmation of histology were sectioned. 5 mice were implanted with four tumors each. The dorsal surface of the mouse was shaved and prepped with betadine solution. A 1cm midline incision was made and with blunt dissection, four pockets were created in four quadrants of the flank of the SCID mouse. One 5mm^2^ tumor implant was placed in each quadrant and the incision was closed with staples.

For SRC implantation, five 3mm^2^ tumor sections were prepared for implantation into five mice, one kidney per mouse. An incision was made in the body wall along the long axis of the kidney. The kidney was gently exposed through the incision, a 4 mm incision was made in the renal capsule, and an implant was inserted. The kidney was gently placed back into the body cavity and incision was closed with chromic gut sutures. For both SQ and SRC implantation, mice were anesthetized using isoflorane with 5% for induction of anesthesia and 1.5% for maintenance. Mice were administered carprofen (7mg/kg, Pfizer) prior to incision to reduce post-operative pain.

For injection into the MFP and IP sties, an adjacent portion of tumor was manually dissociated until fine enough to pass through a 21g needle. Prior to injection, the suspension was added to an equal volume of BD Matrigel (BD Biosciences, Cat#356234), mixed, and injected intraperitoneally (500,000 cells) or into bilateral MFPs (250,000 cells). Five mice were injected IP, and five mice had cells injected into the left and right MFP.

### Treatment of PDX lines with chemotherapy

Once SQ or MFP tumors reached 500 mm^2^ in volume, chemotherapy treatment was initiated in mice from 21 patients. Mice were injected IP with 90 mg/kg of carboplatin and 20 mg/kg of paclitaxel weekly or with vehicle, doses which approximate the maximal tolerated dose used in weekly dose-dense schedule of carboplatin and paclitaxel in patients. Tumors were measured biweekly using calipers. Volume of tumor was calculated using the formula (Length x Width^2^)/2. After 5 weeks of treatment (4 weekly doses, then one week after last chemotherapy dose in order to minimize acute tumor effects of chemotherapy), mice were euthanized by CO­­_2_ asphyxiation and cervical dislocation. Samples of treated and mice treated with vehicle were stored for future analysis. Any remaining tumor was reimplanted for maintenance of the PDX.

### Immunohistochemistry of patient samples and tumors from PDX tumors

Samples in FFPE were cut into 5 μm sections and placed on positively-charged slides. Hematoxylin and eosin stained tissue was analyzed by a gynecologic pathologist to confirm histology. For IHC of ALDH1A1, CD133, CD44, Ki-67 and human-HLA, slides were deparaffinized and rehydrated. Antigen retrieval was with 10 mM sodium citrate at pH 6.0 under pressure. Slides were washed in PBS. Endogenous peroxidases were blocked with 3% H­­_2_O_2_ in methanol. For ALDH1A1, CD133, and CD44, slides were blocked with Ctyo-Q immune-diluent (Innovex Biosciences Cat#NB307) followed by primary antibody incubation in Ctyo-Q immune diluent. Antibody concentrations were as follows: ALDH1A1 – 1:500 (BD Biosciences, Cat#611195) CD133 – 1:500 (Cell Signaling, Cat#3663S), CD44 – 1:500 (Cell Signaling, Cat# 3570S). After primary antibody, slides were washed in PBS. Primary antibody detection was achieved with Mach 4 HRP polymer (Biocare Medical), followed by 3,3'-diaminobenzidine incubation. Slides were counterstained with Gill's Hematoxylin then washed in water and PBS. Slides were sealed with Universal Mount (Open Biosystems, Cat#MBI1232). For Ki-67 (Abgent cat# AJ1427b) and human HLA (Proteintech Group Cat#15240-1), primary antibodies were used at concentrations of 1:200 in 10% normal goat serum. After incubation, slides were washed and blocked with 5% goat serum in 1X PBS. Primary antibody detection was visualized using an anti-rabbit HRP secondary at 1:500 in 5% goat serum (Vector Labs, Cat# PI-1000) and DAB substrate. Slides were counterstained as described above.

### Scoring of IHC for TIC makers and Ki67

Two examiners (AK and CNL) visually estimated the percent of cancer cells staining for ALDH1A1, CD133, CD44, and Ki-67. A 3^rd^ examiner (MGC) was included if there was a >20% discrepancy. The examiners were blinded to the experimental condition for each slide, and a 4^th^ investigator (ZCD) averaged the scores for each specimen and decoded samples for analysis. To be consistent with prior identification of CSCs with flow cytometry, for CD133 and CD44 only expression at the surface membrane was considered. The average number of positive tumor cells for each marker was compared between the untreated PDX tumor and the patient's tumor, and between the treated and untreated PDX, with Student's t-test.

### RT2-qPCR Arrays

RNA extracted from stored samples was converted to cDNA and amplified using the RT^2^ First Strand cDNA Synthesis Kit (SABiosciences). Gene expression was then analyzed using the Cancer Drug Targets RT^2^ Profiler PCR Array (SABiosciences), which profiles the expression of 84 genes that are potential oncogenic targets for anticancer therapeutics [21]. PCR amplification was conducted on an ABI Prism 7900HT and gene expression was calculated using the comparative *C*_T_ method as previously described [43].

### High throughput sequencing of untreated and treated PDX tumors

Sample preparation, raw data prepressing, quality control were conducted in UAB Genomics Core and preliminary analysis was conducted in the UAB Biostatics Core. For RNA-seq, total RNA quality was assessed and the rRNA depleted and concentrated. The RNA-Seq libraries were prepared, validated and quantified. The raw fastq files were aligned to human genome hg19 of a local instance of Partek Flow software package (Saint Louis, MO). Pre-alignment was conducted to determine if trimming is needed based on reads quality score. Aligner STAR was used for best recovery[44]. The BAM files were loaded into Partek Genomics Suite 6.6 (Saint Louis, MO) for further analysis [45]. The reads per kilobase of exon model per million mapped reads (RPKM)-normalized reads were calculated and the expression levels of genes were estimated [46]. Additional filter was applied to exclude genes of low expression. The differential expressions were determined by using paired t-test [47]. Further functional analysis was conducted by using Ingenuity Pathway Analysis (IPA, Redwood City, CA).

## SUPPLEMENTARY MATERIAL, TABLE AND FIGURE


