# Supplementary material for: Using heterogeneity of the patient-derived xenograft model to identify the chemoresistant population in ovarian cancer
Source: Oncotarget. 2014 Aug 19;5(18):8750–64. doi: 10.18632/oncotarget.2373 (PMC4226719; doi:10.18632/oncotarget.2373)
Supplement: Supplementary file 1 [file oncotarget-05-8750-s001.pdf]

Using heterogeneity of the patient-derived xenograft model to identify the chemoresistant population in ovarian cancer

Supplementary Material

Supplementary Table 1: RNAseq revealed 299 genes that had significantly (p<0.05) different expression in the treated PDX samples versus the untreated PDX samples.

Gene	Chromosome	Start Location	Stop Location	Strand	Fold Change (tr/untr)
VSTM4	10	50222290	50323578	-	2.88385
ZNF750	17	80787310	80797932	-	2.44072
RP11-193H5.1	1	238025475	238091620	+	2.38723
ACP5	19	11685475	11689802	-	2.29376
HIST1H2BC	6	26123695	26124133	-	2.14114
CPEB3	10	93808397	94050876	-	2.11674
DNM3	1	171810618	172381858	+	2.02791
MPC1	6	166778408	166796502	-	1.97979
PRPH	12	49688909	49692482	+	1.95466
ABCG1	21	43619799	43717355	+	1.93807
MGLL	3	127407905	127542094	-	1.92425
TLR5	1	223282748	223316625	-	1.88421
MMP14	14	23305742	23316809	+	1.87184
GPC4	X	132435064	132549206	-	1.86717
ITGB2	21	46305868	46348754	-	1.8659
EME2	16	1823229	1826240	+	1.80979
PTK2B	8	27168999	27316909	+	1.77717
FAM219A	9	34398182	34458569	-	1.7753
NMNAT2	1	183217372	183387635	-	1.73956
MOCOS	18	33767480	33848686	+	1.737
PLCB2	15	40580098	40600175	-	1.73671
GCLM	1	94352590	94375013	-	1.73021
ADSSL1	14	105190534	105213648	+	1.72613
LINC00957	7	44078648	44083896	+	1.71886
MKRN9P	12	88176663	88178489	-	1.70392
VAMP2	17	8062465	8066294	-	1.70315
CHST11	12	104850692	105155793	+	1.68861
PTPLAD2	9	21006365	21031636	-	1.68735
ADCY9	16	4012650	4166187	-	1.67735
ZNF727	7	63505821	63538928	+	1.676
PREX1	20	47240793	47444421	-	1.67126
MTSS1	8	125563011	125740749	-	1.66101
HERC2P3	15	20613650	20711434	-	1.65423

NAAA	4	76831808	76862167	-	1.64314
SCN5A	3	38589553	38691165	-	1.63118
MCEE	2	71336806	71357395	-	1.62541
FLCN	17	17115527	17140503	-	1.60288
HNRNPUL2-BSCL2	11	62457734	62494857	-	1.59257
ABCA7	19	1040102	1065571	+	1.58868
ZNF486	19	20278023	20311300	+	1.57838
ETV3	1	157094459	157108384	-	1.57332
TMEM200C	18	5890184	5892104	-	1.57315
UQCRHL	1	16133657	16134195	-	1.57235
RNASEL	1	182542769	182558395	-	1.55592
HTATIP2	11	20385231	20405330	+	1.55223
IER5	1	181057638	181059980	+	1.55116
RPRML	17	45055522	45056615	-	1.53476
TSSK6	19	19625028	19626470	-	1.53236
ANKRD18A	9	38571361	38620361	-	1.52864
TIMP2	17	76849059	76921473	-	1.52806
RASD1	17	17397753	17399710	-	1.5132
MYZAP	15	57884102	57977563	+	1.50081
STAG3L5P	7	99933702	99938952	+	1.4968
HPCA	1	33352098	33360248	+	1.48945
HSF4	16	67197288	67203849	+	1.47828
STIM1	11	3876933	4114441	+	1.45092
GNAI1	7	79764140	79848726	+	1.44357
MAPK7	17	19281034	19286858	+	1.44139
APOOP5	16	59788045	59789096	-	1.43991
HLA-A	6_ssto_hap7	1150098	29913662	+	1.43605
FAM84A	2	14772810	14780169	+	1.42164
SYNM	15	99645286	99675801	+	1.41178
PKIA	8	79428336	79517503	+	1.40459
STARD13	13	33677272	34250933	-	1.40261
DYX1C1-CCPG1	15	55647421	55790783	-	1.40222
EYA3	1	28296855	28415149	-	1.3939
MNT	17	2287354	2304259	-	1.38268
PRKAR2A	3	48788093	48885271	-	1.38138
SLC25A28	10	101370275	101380222	-	1.37891
SERINC3	20	43124864	43150727	-	1.37137
BLVRB	19	40953691	40971726	-	1.36946
ADIPOR1	1	202909960	202927701	-	1.35143
SEC22B	1	145096407	145116998	+	1.34893
PHF12	17	27232271	27278509	-	1.34879

KIAA1614	1	180882313	180915240	+	1.34765
ZDHHC1	16	67428322	67450340	-	1.34184
DYNLL2	17	56160780	56167619	+	1.33573
MFSD1	3	158519715	158547509	+	1.33549
KBTBD4	11	47593749	47600568	-	1.32875
ZBTB43	9	129567285	129600488	+	1.32686
ENTPD5	14	74433181	74486027	-	1.32495
C14orf142	14	93669237	93673460	-	1.32027
CHMP5	9	33264877	33282068	+	1.31782
RNF139	8	125487008	125500860	+	1.3174
DIRC2	3	122513901	122599987	+	1.31647
MID1IP1	X	38660685	38665784	+	1.30884
TM9SF2	13	100153628	100216303	+	1.30783
PLEKHM1	17_ctg5_hap1	128328	43568147	-	1.30503
ATP1B3	3	141595470	141645383	+	1.30497
PPT2	6_ssto_hap7	3382242	32131459	+	1.30378
CLIC2	X	154505496	154563991	-	1.30032
PRPF8	17	1553923	1588177	-	1.29751
TOM1	22	35695268	35743988	+	1.29725
PYGB	20	25228706	25278649	+	1.29328
ESYT3	3	138153415	138197257	+	1.29239
FAM129B	9	130267617	130341287	-	1.29145
PSMD1	2	231921578	232037541	+	1.28958
GABARAPL2	16	75600249	75611780	+	1.28721
ESRRA	11	64073000	64084213	+	1.28529
MEF2D	1	156433513	156470635	-	1.2823
TMBIM1	2	219138917	219157281	-	1.28031
CALCOCO2	17	46908350	46942608	+	1.2781
GOSR2	17	45000486	45018734	+	1.27738
S100A6	1	153507076	153508718	-	1.27694
WDR81	17	1619817	1641894	+	1.27622
FAM89A	1	231154704	231175996	-	1.27382
MCOLN1	19	7587496	7598896	+	1.27292
MAP3K10	19	40697651	40721483	+	1.27281
ZNF319	16	58028573	58033763	-	1.26794
ZAK	2	173940565	174132738	+	1.2671
OXNAD1	3	16306667	16347595	+	1.26545
TECPR1	7	97844755	97881564	-	1.26021
KLHL36	16	84682131	84695917	+	1.25481
LPIN3	20	39969560	39989223	+	1.24997
STAG3L2	7	74298092	74306732	-	1.24744

GPR137	11	64051811	64056973	+	1.23724
H2AFZ	4	100869244	100871513	-	1.23068
FAM27E3	9	67784944	67786626	-	1.2279
AFAP1L2	10	116054583	116164538	-	1.22724
RIN3	14	92980125	93155335	+	1.22721
KIF17	1	20990507	21044511	-	1.22185
SEC24C	10	75504131	75531934	+	1.21563
HECTD3	1	45468220	45477028	-	1.21411
COPA	1	160258377	160313355	-	1.21305
RSBN1L-AS1	7	77313168	77326663	-	1.20939
SQSTM1	5	179233388	179265078	+	1.20887
KLHL18	3	47324330	47388307	+	1.20439
ZNF555	19	2841433	2860473	+	1.20269
BCAS3	17	58755172	59470200	+	1.20016
KLC4	6	43027372	43042834	+	1.19962
PTPDC1	9	96793076	96872139	+	1.19953
C14orf37	14	58470808	58618848	-	1.19452
RXRA	9	137218316	137332432	+	1.19415
FTO	16	53737875	54148380	+	1.19311
LOC344967	4	40044537	40058820	-	1.19245
TLDC1	16	84509966	84538289	-	1.19096
CLPX	15	65442784	65477564	-	1.18712
YIPF5	5	143537723	143550279	-	1.18286
ENDOD1	11	94822974	94865816	+	1.18088
SURF4	9	136228325	136244821	-	1.1789
SCAMP2	15	75137197	75165671	-	1.17767
PSAP	10	73576055	73611083	-	1.17538
SLC25A44	1	156163723	156182588	+	1.16728
RAB7A	3	128444979	128533642	+	1.16442
ATXN7	3	63850233	63989137	+	1.15919
NUFIP2	17	27582854	27621167	-	1.15842
MKRN1	7	140152840	140179370	-	1.15465
LOC254896	8	22941868	22961071	+	1.14851
PIAS1	15	68346572	68480405	+	1.14763
RSPRY1	16	57220241	57272948	+	1.14671
HSPA9	5	137890571	137911319	-	1.1462
SKIV2L	6_ssto_hap7	3212164	31937533	+	1.13908
OSBP	11	59341871	59383618	-	1.13833
EMC3	3	10005636	10028523	-	1.13652
THAP6	4	76439654	76455237	+	1.13453
STX12	1	28099694	28150964	+	1.11803

HCCS	X	11129406	11141205	+	1.11749
TAF12	1	28929609	28969605	-	1.11704
MESDC1	15	81293295	81296346	+	1.11191
GADD45G	9	92219927	92221470	+	1.10348
RHOA	3	49396579	49449527	-	1.08749
ASTN1	1	176826441	177134041	-	1.08508
ECD	10	74894282	74927854	-	1.07666
LAMTOR3	4	100799495	100815704	-	-1.08305
RNF44	5	175953700	175964422	-	-1.08391
HNRNPDL	4	83343717	83351379	-	-1.09082
ZNF407	18	72342919	72777629	+	-1.09614
MAP7D3	X	135295379	135338642	-	-1.1053
LOC100129361	12	11323780	11328620	+	-1.12191
DZANK1	20	18364011	18447830	-	-1.13122
WWC2	4	184020463	184241930	+	-1.13131
ING5	2	242641456	242668897	+	-1.13132
GIT2	12	110367607	110434195	-	-1.13264
TM2D3	15	102182049	102192595	-	-1.13901
RUSC1-AS1	1	155290251	155293939	-	-1.13993
NADK2	5	36192691	36242382	-	-1.14161
RTEL1	20	62289163	62327607	+	-1.14575
TCEAL8	X	102507923	102510122	-	-1.14941
SPDL1	5	169010638	169031782	+	-1.15387
ZCCHC8	12	122956146	122985621	-	-1.16359
MDH1B	2	207602489	207630274	-	-1.16858
IFNGR2	21	34775202	34809829	+	-1.16925
CXorf23	X	19930980	19988383	-	-1.17025
FRG1	4	190861974	190884360	+	-1.1777
ZSCAN9	6	28193029	28201265	+	-1.18814
USP42	7	6144550	6201196	+	-1.18876
ATG12	5	115163894	115177549	-	-1.19193
CARF	2	203776941	203851209	+	-1.19386
HIST1H1C	6	26055968	26056700	-	-1.19716
PHC1	12	9067316	9094061	+	-1.19765
SECISBP2L	15	49280835	49338761	-	-1.2011
TOP2B	3	25639396	25705864	-	-1.20271
PIGB	15	55611133	55647847	+	-1.2065
GATAD1	7	92076762	92089382	+	-1.20707
PPHLN1	12	42719947	42842423	+	-1.20845
RBM19	12	114254543	114404177	-	-1.21448
CCDC88C	14	91737667	91884189	-	-1.21455

RNF138	18	29671818	29711525	+	-1.21865
MNAT1	14	61201459	61435399	+	-1.2339
FJX1	11	35639735	35642422	+	-1.23581
C1orf52	1	85715637	85725356	-	-1.23745
ZNF169	9	97021578	97065292	+	-1.24774
N6AMT1	21	30244513	30257696	-	-1.24812
LINC00998	7	112756773	112758638	-	-1.25062
UCK2	1	165796732	165880856	+	-1.25073
RSL1D1	16	11928055	11945443	-	-1.25096
RAB3IP	12	70132466	70216985	+	-1.25528
APAF1	12	99039078	99129212	+	-1.25549
TEKT4P2	21	9907189	9968594	-	-1.25804
POLR1C	6	43484777	43489247	+	-1.25846
ZNF74	22	20748405	20762754	+	-1.25997
HIST1H2BK	6	27106072	27114638	-	-1.26072
NPM1	5	170814708	170837889	+	-1.26122
EIF3C	16	28390903	28747051	+	-1.26145
NPHP3	3	132399453	132441304	-	-1.26174
DLG1	3	196769431	197025448	-	-1.26528
HCG8	6_ssto_hap7	1219726	29981700	-	-1.26818
TDRD3	13	60970591	61148014	+	-1.27252
WARS2	1	119573839	119683296	-	-1.2741
PHKA2	X	18910416	19002481	-	-1.27564
ALG10B	12	38710557	38723529	+	-1.27866
IDI1	10	1085964	1095062	-	-1.27877
PRKD2	19	47177573	47220385	-	-1.28338
ANAPC7	12	110810705	110841536	-	-1.28362
NEDD4L	18	55711610	56068773	+	-1.28409
IPO8	12	30781915	30848930	-	-1.28571
ZNHIT6	1	86115106	86174117	-	-1.29108
CROCC	1	17248445	17299475	+	-1.29865
NACA	12	57106211	57119327	-	-1.30339
MTX3	5	79272539	79287089	-	-1.30457
PTRHD1	2	25013136	25016252	-	-1.31352
TTC33	5	40711678	40756073	-	-1.31977
LRRC40	1	70610485	70671362	-	-1.32137
CCDC14	3	123632274	123680256	-	-1.32234
SUDS3	12	118814358	118855841	+	-1.33179
ZNF140	12	133657037	133684259	+	-1.33382
NDUFA5	7	123181083	123197959	-	-1.33767
PPA2	4	106290234	106395228	-	-1.33974

DCBLD2	3	98514814	98620534	-	-1.34346
IMMP2L	7	110303106	111202574	-	-1.34688
COMMD10	5	115420727	115628979	+	-1.35281
SEPHS1	10	13359438	13390299	-	-1.35534
NFATC2	20	50003494	50179371	-	-1.35748
CEP135	4	56814974	56899530	+	-1.3608
TMEM220	17	10616639	10633647	-	-1.36411
PGM2L1	11	74041361	74109503	-	-1.37177
L3MBTL3	6	130339728	130462595	+	-1.37235
TMA16	4	164415673	164441692	+	-1.3728
WDR77	1	111982512	111991831	-	-1.37912
LBR	1	225589204	225616558	-	-1.38056
NLN	5	65018023	65125112	+	-1.38189
FUT1	19	49251268	49258648	-	-1.38278
SERF1B	5	69321078	70214352	+	-1.38545
C12orf45	12	105380098	105388506	+	-1.38697
ADAM1A	12	112336867	112339707	+	-1.38905
NFKBIE	6	44225903	44233526	-	-1.38947
DPH5	1	101455180	101491363	-	-1.39075
ZNF681	19	23921997	23941694	-	-1.39198
TSHZ1	18	72922710	73001906	+	-1.39363
PRKCI	3	169940220	170023771	+	-1.40073
HSPG2	1	22148737	22263751	-	-1.40283
LONRF1	8	12579406	12612993	-	-1.40439
FAM161A	2	62051983	62081279	-	-1.40476
MCOLN2	1	85391266	85462797	-	-1.41279
C12orf60	12	14956506	14976792	+	-1.41351
ADM5	19	50191942	50194248	+	-1.41816
F2RL1	5	76114833	76131141	+	-1.41876
RSL24D1	15	55473512	55489232	-	-1.41939
TMEM183A	1	202976534	202993198	+	-1.42322
RTKN2	10	63952845	64028623	-	-1.43339
GLB1L2	11	134201768	134246219	+	-1.44439
ZNF596	8	182137	197341	+	-1.44515
PCDHB16	5	140561265	140565797	+	-1.4581
LOC100133091	7	76178658	76257300	+	-1.46254
DTWD1	15	49913226	49937334	+	-1.46696
SGTB	5	64961755	65017942	-	-1.46797
TFAP4	16	4307187	4323002	-	-1.47735
CAPS	19	5914193	5916223	+	-1.48887
FBRSL1	12	133067157	133161774	+	-1.49516

CHRNA10	11	3686817	3692615	-	-1.51081
ALKBH2	12	109525993	109531294	-	-1.51986
FAM86A	16	5134301	5147790	-	-1.52015
LOC100506548	5	40825365	40829245	-	-1.52016
SYCE1L	16	77233349	77246977	+	-1.53511
BEND3	6	107386385	107435637	-	-1.54651
DGKA	12	56324946	56347808	+	-1.54827
GPR125	4	22388997	22517678	-	-1.55067
KLF5	13	73629114	73651681	+	-1.55448
CKAP4	12	106631659	106641714	-	-1.56377
MECOM	3	168801287	169381564	-	-1.56982
ARNT2	15	80696692	80890278	+	-1.60687
FAM133B	7	92190072	92219709	-	-1.61605
PUS7	7	105096960	105162686	-	-1.61787
PLCE1	10	95753746	96088149	+	-1.73754
TES	7	115850547	115898838	+	-1.75919
POLR3G	5	89770681	89810370	+	-1.86241
EGFL8	6_ssto_hap7	3393402	32136063	+	-2.09768
GPHA2	11	64701943	64703361	-	-2.26238
APOC1	19	45417921	45422607	+	-2.48786

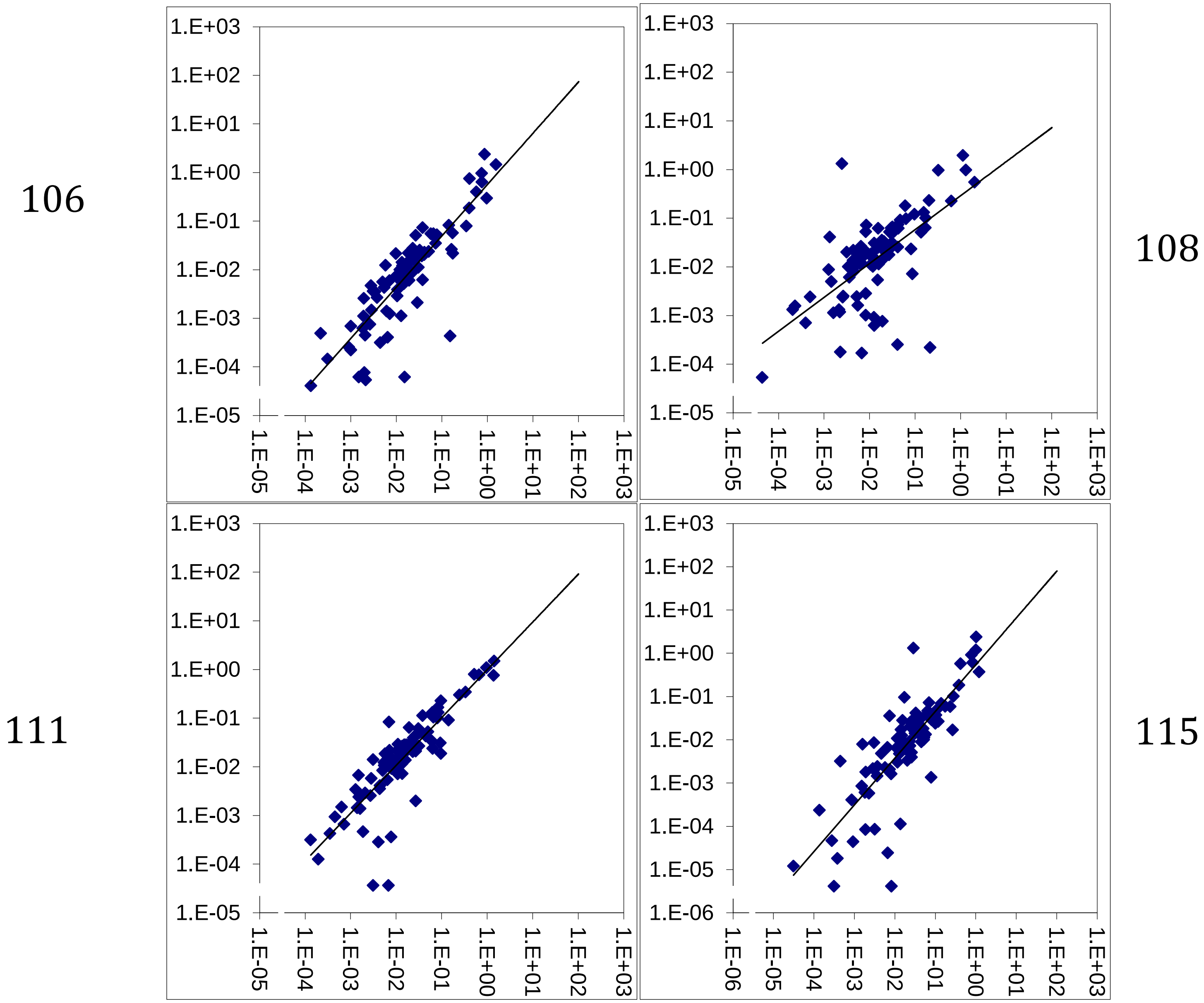

Supplementary Figure 1: The SABiociences RT^2 qPCR array for cancer drug targets was run on the patient's tumor and their matched untreated PDX tumor. Differences in relative gene expression for each target was calculated and the $2^{\Delta Ct}$ value was determined. Correlation of expression is seen in each of the 4 pairs analyzed.
